# Protein Changes in Shade and Sun *Haberlea rhodopensis* Leaves during Dehydration at Optimal and Low Temperatures

**DOI:** 10.3390/plants12020401

**Published:** 2023-01-15

**Authors:** Gergana Mihailova, Ádám Solti, Éva Sárvári, Éva Hunyadi-Gulyás, Katya Georgieva

**Affiliations:** 1 Institute of Plant Physiology and Genetics, Bulgarian Academy of Sciences, Acad. Georgi Bonchev Str., Bl. 21, 1113 Sofia, Bulgaria; 2 Department of Plant Physiology and Molecular Plant Biology, Institute of Biology, Eötvös Loránd University, Pázmány P. Sétány 1/C, H-1117 Budapest, Hungary; 3 Laboratory of Proteomics Research, Biological Research Centre, Eötvös Loránd Research Network, Temesvári Krt. 62., H-6726 Szeged, Hungary

**Keywords:** drought stress, frost-induced desiccation, LC-MS/MS, proteomics, resurrection plants

## Abstract

*Haberlea rhodopensis* is a unique resurrection plant of high phenotypic plasticity, colonizing both shady habitats and sun-exposed rock clefts. *H. rhodopensis* also survives freezing winter temperatures in temperate climates. Although survival in conditions of desiccation and survival in conditions of frost share high morphological and physiological similarities, proteomic changes lying behind these mechanisms are hardly studied. Thus, we aimed to reveal ecotype-level and temperature-dependent variations in the protective mechanisms by applying both targeted and untargeted proteomic approaches. Drought-induced desiccation enhanced superoxide dismutase (SOD) activity, but FeSOD and Cu/ZnSOD-III were significantly better triggered in sun plants. Desiccation resulted in the accumulation of enzymes involved in carbohydrate/phenylpropanoid metabolism (enolase, triosephosphate isomerase, UDP-D-apiose/UDP-D-xylose synthase 2, 81E8-like cytochrome P450 monooxygenase) and protective proteins such as vicinal oxygen chelate metalloenzyme superfamily and early light-induced proteins, dehydrins, and small heat shock proteins, the latter two typically being found in the latest phases of dehydration and being more pronounced in sun plants. Although low temperature and drought stress-induced desiccation trigger similar responses, the natural variation of these responses in shade and sun plants calls for attention to the pre-conditioning/priming effects that have high importance both in the desiccation responses and successful stress recovery.

## 1. Introduction

Resurrection plants represent a small group of angiosperms that possess the ability to survive desiccation to an air-dry state and recover to normal metabolic functions upon rehydration [1]. *Haberlea rhodopensis,* like other European species such as *Ramonda serbica, Ramonda nathaliae, Ramonda myconi,* and *Jankaea heldreichii*, is a desiccation-tolerant member of the Gesneriaceae family [2,3,4,5]. The homoiochlorophyllous resurrection plant *H. rhodopensis* is a Tertiary relict on the Balkan Peninsula [6,7]. In its natural habitat in the Rhodope Mountains, *H. rhodopensis* colonizes rock surfaces at an altitude from 136 to near 1600 m a.s.l., and the sites of occurrence extremely differ in temperature, humidity, and light conditions [8]. Although the taxon has a clear preference for shady habitats (e.g., north-faced limestone or undercanopy silicate rocks and maximum photosynthetically active photon flux density (PPFD) of 25–30 µmol m^−2^ s^−1^; these are referred to as shade plants), *H. rhodopensis* also colonizes rock clefts directly exposed to sunlight (maximum PPFD of 1500–1700 µmol m^−2^ s^−1^; referred to as sun plants). In the natural habitat, both shade and sun plants undergo desiccation in response to drought stress due to the lack of precipitation during the summer [9,10]. Moreover, in contrast to most resurrection plants of tropical/subtropical origin, *H. rhodopensis* also survives freezing temperatures (below −20 °C) during the winter [8,11]. Overwintering also induces desiccation mechanisms [11].

Drought stress inhibits photosynthesis in leaves by affecting the stomatal opening and closing and by the induction of oxidative stress [12]. Since *H. rhodopensis* is a homoiochlorophyllous resurrection plant, chlorophyll molecules continue to absorb the energy of light under drought. Although effective non-photochemical quenching mechanisms develop during the desiccation [13], inhibition of carbon assimilation contributes to the transfer of the reducing capacity to O_2_, forming reactive oxygen species (ROS) [14]. ROS can damage nucleic acids, carbohydrates, lipids, and proteins, among other components; thus, antioxidative protection has a primary importance. Resurrection plants apply various mechanisms including cell wall and membrane modifications, accumulation of osmolytes/compatible solutes, antioxidants and ROS-scavenging enzymes, and multiple types of protective proteins to restrain structural damages and protect their metabolism from the detrimental effect of ROS [15].

Stabilization of macromolecules at low relative water content (RWC) when water loss occurs is of great importance in resurrection plants [16]. A significant role for cell protection in response not only to dehydration but also to different stress factors is played by proteins such as dehydrins, small heat shock proteins (sHSPs), and early light-induced proteins (ELIP) [17,18]. Dehydrins accumulate in response to dehydration caused by water stress, salt stress, low or high temperatures, and heavy metal toxicity [19,20]. They have chaperone-like functions in plant cells related to the protection of proteins and membrane stabilization during stress, but they also work as ROS scavengers [21]. Their mechanism of functioning is still not well understood [21,22]. Plant sHSPs are part of the HSP superfamily, and they are constitutively expressed in plant cells at low concentrations and strongly induced in response to different types of stress. They act as ATP-independent molecular chaperones preventing the irreversible aggregation of denatured proteins [23]. Among living beings, plants possess the most numerous group of sHSPs, some of them accumulating more than 40 different sHSPs [23].

In the lack of effective photosynthesis, the carbohydrate metabolism of autotrophic plant cells is shifted towards catabolism and cell-decomposing (autophagy) processes. During desiccation, the carbon assimilation decays in *H. rhodopensis* [24,25], since Calvin cycle enzymes stay under a fundamental redox regulation that switches out the function, primarily that of glyceraldehyde-3-phosphate dehydrogenase and phosphoribulokinase, caused by the lack of sufficient reducing power in the chloroplast thioredoxin system [26,27]. Nevertheless, the decreased production of reducing power and ATP by the photosynthetic electron transport chain increases the need for substrate-level phosphorylation and glucose oxidation in the catabolic metabolism [28]. However, *H. rhodopensis* performs a complete remodeling of the cellular sugar composition during the desiccation processes and accumulates sucrose, among other molecules, together with the formation of secondary vacuoles that are thought to be the sites for sucrose storage in the mesophyll cells in the desiccated stage [29]. Rearrangement of the cell constituents requires massive biosynthetic and movement actions, the bioenergetics background of which has not been revealed so far.

In previous studies, we have indicated that *H. rhodopensis* sun plants are hardier in multiple aspects against stresses related to desiccation [9,10,13,30]. Although the phenology of shade and sun plants shows high variation among the ecotypes, the ultrastructure and the organization of thylakoids were found to be similar in both populations [9]. Sun plants showed higher rates of CO_2_ assimilation and higher PSII and PSI activity not only in the well-hydrated state but also during dehydration [10,30]. In response to dehydration, sun plants were shown to apply light-harvesting antennae-based non-photochemical quenching mechanisms to dissipate excess energy in contrast to the shade plants that mainly perform a re-emission of excitation energy from inactivated PSII reaction centers [10,13]. The abundances of *β*-carotene and xanthophyll cycle pigments were indicated to be higher in shade plants than in sun ones [10]. While the changes in gene expression occurred earlier in sun plants [30], the amount of main thylakoid proteins, including Rubisco large subunit, decreased strongly in response to drought stress in shade plants [29,31]. Altogether, desiccation-induced responses were more pronounced in sun plants which also possessed a greater capacity to recover after rehydration [9,10,30,31]. In the present study, we aimed to reveal ecotype-level and temperature-dependent variations that enable desiccation tolerance by applying both targeted and untargeted proteomic approaches.

## 2. Results

### 2.1. Stress-Induced Changes in the Leaf Proteome

**Figure 1:**
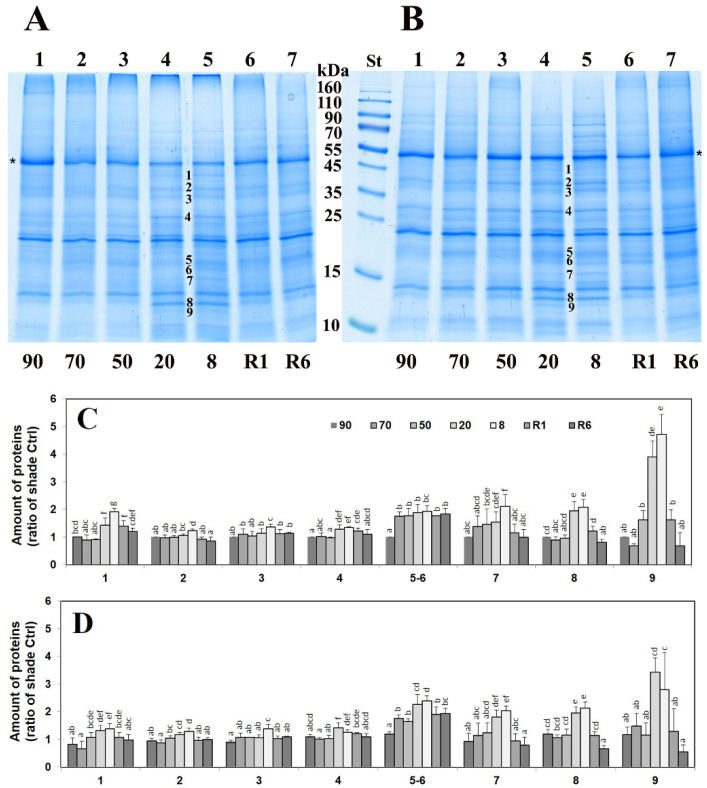
SDS PAGE pattern of total leaf polypeptides in *H. rhodopensis* shade (**A**) and sun (**B**) control plants (90% RWC), during dehydration (70, 50, 20, and 8% RWC) and after rehydration (1 and 6 days of rehydration; R1, RWC 50%, and R6, RWC 90%, respectively). (**A**,**B**) Coomassie Brilliant Blue-stained polypeptide patterns of controls (lanes 1), dehydrated leaves (lanes 2–5), and rehydrated ones (lanes 6 and 7). Approx. 10 μg protein was applied per lane. St: Fermentas Page Ruler Prestained Protein SM0671 (Thermo Fisher Scientific, Waltham, MA, USA) standards. (**C**,**D**) Changes in the amount of the elevating leaf polypeptides numbered in (**A**,**B**) (1–9) in shade (**C**) and sun (**D**) ecotypes of *H. rhodopensis*. The relative protein amounts (pixel density of the protein bands; arbitrary unit) of numbered bands were expressed as the percentage of summa pixel density of lanes; for better comparison, values of each protein were normalized so that control samples of shade ecotype (90% RWC) were chosen as 1. Values are given as mean ± SD (*n* = 3). Changes between shade and sun plants were statistically compared. Different letters within a graph indicate significant differences assessed by the Fisher LSD test (*p* ≤ 0.05) after performing multifactor ANOVA. Asterisks (*) on (**A**,**B**) show the position of Rubisco large subunit on the gels.

We compared the 1D polyacrylamide gel electrophoresis (PAGE) patterns of total protein extracts of shade and sun *H. rhodopensis* leaves during desiccation and exposure to freezing stress as well as after recovery of the plants. It is important to note that both drought and freezing stresses trigger the dehydration of *H. rhodopensis* plants (Figure 1 and Figure S1). Dehydration induced by both drought and low-temperature stresses resulted in the increased density of several bands in the same regions compared to unstressed *H. rhodopensis* shade plants (referred to as “control”) which returned to the level of control after rehydration (Figure 1A, Figure S1A and S2). The molecular masses of these bands were calculated to be around 48 (1), 41 (2), 38 (3), 31 (4), 19 (5), 18 (6), 17 (7), 14 (8), and 13.5 (9) kDa. Changes in the PAGE band densities were similar in the leaves of sun plants compared to shade ones (Figure 1B and Figure S1B). The increase in the abundance of the detected bands was more pronounced under drought than the low-temperature-induced dehydration (Figure S2). Under the drought-induced desiccation, the elevated abundance of the bands became significant under 50% RWC, and the band intensity returned to the control level after 6 days of rehydration (Figure 1C,D). Exposure to low but above-zero temperatures did not significantly change the PAGE pattern of the total leaf proteins. The rise in the abundance of the PAGE bands only started when the dehydration process was initiated by low temperatures and was the most pronounced under 0 °C (lines 4 and 5 in Figure S1A and line 3 in Figure S1B), when the RWC of leaves decreased to 50–60%.

### 2.2. Activity of SOD Isoenzymes

**Figure 2:**
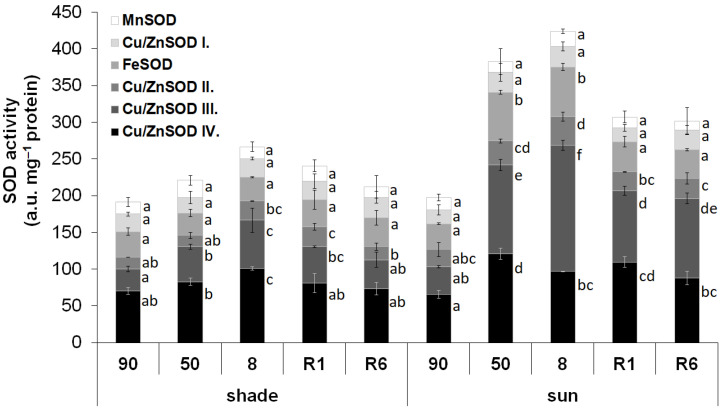
Stacked column plot of the activity of superoxide dismutase (SOD) isoenzymes in the leaves of shade and sun *H. rhodopensis* plants. Total SOD activity (represented by the total height of the columns) is divided into the activity of SOD isoenzymes based on native polyacrylamide gel electrophoresis. Activities were measured in controls (90% RWC) and during the stages of dehydration (50 and 8% RWC) and rehydration (1 and 6 days of rehydration; R1 and R6, respectively). To compare the differences within the corresponding isoenzyme activities, one-way ANOVAs were performed with Tukey–Kramer post hoc test on the SOD isoenzymes (*p* < 0.05; *n* = 5).

SOD isoforms were separated by native polyacrylamide gel electrophoresis. The activity of the isoforms was detected by in-gel activity staining. Both in shade and sun *H. rhodopensis* leaves, the activity of six SOD isoenzymes was detected at R_f_ of 0.170, 0.203, 0.270, 0.323, 0.372, and 0.412 (Figure S3). Based on literature evidence [32], these isoenzymes were identified as MnSOD, Cu/ZnSOD I, FeSOD, Cu/ZnSOD II, Cu/ZnSOD III, and Cu/ZnSOD IV, respectively. The SOD activity in the leaves of not-stressed shade and sun plants was similar (Figure 2). Desiccation induced a significant increase in the SOD activity in both plant types, but this induction was higher in the leaves of sun plants (115.0% of the well-hydrated leaves) than in the shade plants (39.6% of the well-hydrated leaves). The activity of all SOD isoenzymes was increased by dehydration in the leaves of both shade and sun plants. The higher induction of the SOD activity in the sun-exposed plants was based on the increased activity of FeSOD and Cu/ZnSOD III. It is important to note that the activity of FeSOD did not show a significant induction in response to the desiccation of leaves of the shade plants. The activities of other SOD isoenzymes showed similar increases upon desiccation in the leaves of both sun and shade plants.

### 2.3. Protective Proteins

**Figure 3:**
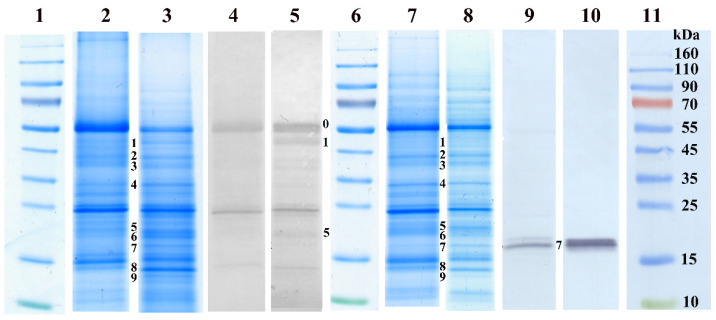
Changes in the leaf polypeptide patterns and in the density of dehydrin bands of *H. rhodopensis* shade (lanes 2–5) and sun plants (lanes 7–10) under drought stress. Polypeptide patterns of control (lanes 2, 4, 7, and 9, RWC 80–90%) and dried leaves (lanes 3, 5, 8, and 10, RWC 8%) either stained (lanes 2, 3, 7, and 8) or blotted against dehydrin (lanes 4 and 5) and sHSPs (lanes 9 and 10). Bands of increased intensity under the stress are numbered next to the Western blots. Lanes 1, 6, and 11: Fermentas Page Ruler Prestained Protein SM0671 (Thermo Fisher Scientific, Waltham, MA, USA) standards.

**Figure 4:**
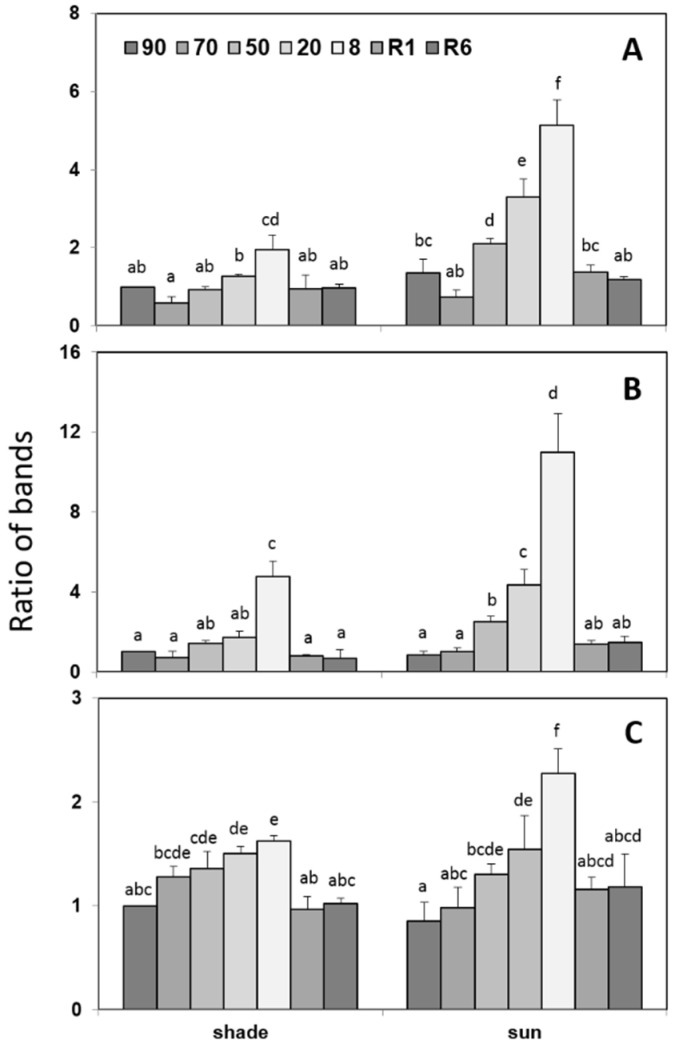
Changes in the density of leaf dehydrin bands 0 (**A**), 1 (**B**), and 5 (**C**) in the shade and sun *H. rhodopensis* plants in controls (90%), during dehydration (70, 50, 20, and 8% RWC) and after rehydration (1 and 6 days of rehydration; R1 and R6, respectively). Amounts of dehydrins were determined by Western blotting and normalized to the same total stained protein values in the lanes. For better comparison of the kinetics of changes in a given polypeptide in the shade and sun leaves, shade control values were chosen as 1. Values are given as mean ± SD (*n* = 4). Changes between shade and sun plants were statistically compared. Different letters within a graph indicate significant differences assessed by the Fisher LSD test (*p* ≤ 0.05) after performing multifactor ANOVA.

Accumulation of dehydrins and sHSPs during desiccation of *H. rhodopensis* leaves was monitored by Western blot using specific antibodies. In Figure 3, we demonstrate the differences between control and desiccated leaves by PAGE pattern and immunoblot signals using anti-dehydrin and anti-sHSP antibodies, respectively. Immunoblot analysis with anti-dehydrin antibodies of control and desiccated shade leaves (8% RWC) indicated several bands in the molecular mass range of 14–57 kDa (Figure 3, lanes 4 and 5). In the dehydrated leaves, the increase in the signal was the most pronounced in bands 0, 1, and 5 (57, 48, and 19 kDa) compared to the control (Figure 3, line 5 versus line 4). Quantitative changes of dehydrins during dehydration and after rehydration of the shade and sun *H. rhodopensis* plants are presented in Figure 4 (see also Figure S4). Slight and moderate dehydration (70–50% RWC) did not affect the accumulation of dehydrins significantly, but their amounts, especially those of bands 0 and 1, sharply increased in severely dehydrated and desiccated leaves (20% and 8% RWC). Rehydration decreased the quantity of the detected dehydrins close to the level detected in the control. The accumulation of dehydrins during dehydration of *H. rhodopensis* was more pronounced in the leaves of sun plants compared to the shade ones (Figure 4).

**Figure 5:**
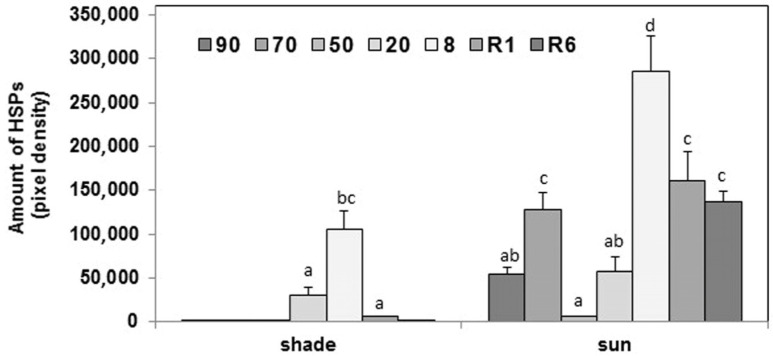
Changes in the relative amounts of total sHSPs in leaves of shade and sun *H. rhodopensis* plants during dehydration and rehydration. The relative amounts of the sHSP bands were determined based on Western blotting and normalized to the same total stained protein values in the lanes. Values are given as mean ± SD (*n* = 3). Changes between shade and sun plants were statistically compared. Different letters within a graph indicate significant differences assessed by the Fisher LSD test (*p* ≤ 0.05) after performing multifactor ANOVA.

Western blots against sHSPs detected only one major band (~17 kDa) and two faint bands of similar molecular weights to the major one in *H. rhodopensis* leaves (Figure 3, lanes 9 and 10). In the sun plants, sHSPs were present in the control leaves, and during dehydration, their relative amounts increased, especially in fully desiccated plants (Figure 5 and Figure S5). As a response to rehydration, the relative amounts of sHSPs started to decrease but still remained higher at R6 compared to the control. In the shade *H. rhodopensis* leaves, however, sHSPs were only detected in severely dehydrated and desiccated leaves (20% and 8% RWC). The sHSP bands almost disappeared after 1 day of rehydration (R1). The changes in the amounts of sHSPs during dehydration, such as the accumulation of dehydrins, were much more remarkable in the leaves of sun plants compared to shade ones.

### 2.4. Untargeted Detection of Changes in the Proteome

In order to identify further stress-induced proteins, polypeptide bands of total leaf protein patterns that changed in intensity strongly during desiccation and rehydration were subjected to LC-MS/MS detection and peptide identification. These polypeptide bands (1 and 8) were cut from identical regions of control and completely desiccated leaves (8% RWC) of *H. rhodopensis* shade plants (Figure S6).

Hits of the alignment of the achieved peptide sequences (Table S1) by LC-MS/MS against the closest relative (*Dorcoceras hygrometricum* Bunge syn. *Boea hygrometrica* (Bunge) R.Br., Gesneriaceae, Oleales) (Table S2) were further analyzed based on the comparison of the predicted molecular weights of the peptides and the detected molecular weights of the bands of interest. Weak hits as well as peptides that most likely came from protein degradation (predicted molecular weight is significantly higher than that of the detected band) were excluded from the analysis. Since a 1D SDS PAGE band is necessarily a composite of multiple polypeptides due to the low resolution of the 1D separation, the analysis of the increased density of the bands cannot be translated to the changes in the relative abundance of the identified proteins. Thus, by comparing the polypeptides revealed in the bioinformatical analysis, proteins that were absent in the control but present in the desiccated leaves were further analyzed as proteins for which the abundance has surely changed upon desiccation. The analysis resulted in the identification of six polypeptides in Band 1 and five of them in Band 8 that were absent in the corresponding band of the control but present in that of the desiccated leaves (Table 1). In Band 1 (approx. 48 kDa), we identified enolase, UDP-D-apiose/UDP-D-xylose synthase 2, V-type proton ATPase (VHA) subunit H, a 55 kDa protein of unknown function (F511_06435), a 52 kDa protein of unknown function (F511_00655), and a 47 kDa protein of unknown function (F511_12006). In Band 8 (approx. 14 kDa), triosephosphate isomerase, early light-induced protein (ELIP), pectin methylesterase (fragment), galactose mutarotase (fragment), and a 15 kDa protein of unknown function were detected. To assume the function of the proteins of unknown function (hypothetical proteins), *D. hygrometricum* protein sequences were subjected to reverse blasting against Viridiplantae protein sequences (Table S3). Analysis indicated that the 55 kDa hypothetical protein shares high similarity to 81E8-like cytochrome P450 monooxygenases. Blast results indicated that the 52 kDa hypothetical protein is a Tu-class elongation factor in the protein translation of organelles/chloroplasts, while the 47 kDa hypothetical protein functions as a 4A class initiation factor in the eukaryotic type of protein translation/mRNA binding. The function of the 15 kDa hypothetical protein cannot be directly revealed from blast results since the function of the highest coverage/identity hits has not come out. Nevertheless, according to the reverse blast result, the protein belongs to a drought stress-induced group of the vicinal oxygen chelate metalloenzyme (1^VOC^) superfamily (Table S3).

Therefore, proteins that were only identified in desiccated leaves can be clustered in six different categories: carbohydrate metabolism (enolase, UDP-D-apiose/UDP-D-xylose synthase 2; triosephosphate isomerase; pectin methylesterase, galactose mutarotase), protein biosynthesis (Tu-class elongation factor, 4A class initiation factor), tonoplast proteins (VHA subunit H), oxygenases (81E8-like cytochrome P450 monooxygenase), early light stress-induced proteins (ELIP), and desiccation-related proteins (1^VOC^ superfamily protein).

## 3. Discussion

*Haberlea rhodopensis* is the second most investigated taxon among resurrection plants after the tropical *Craterostigma plantagineum* according to the number of published studies so far [33], but proteome-level investigations, in particular on the stress-induced proteins, are scarce. In this study, we applied targeted and untargeted analyses related to dehydration including shade and sun ecotypes of *H. rhodopensis*. We also considered low-temperature-induced dehydration, a mechanism that proved to share high similarities to drought-induced desiccation in *H. rhodopensis* [11].

Accumulation of stress-induced proteins is among the main defense mechanisms in resurrection plants upon drought stress [34]. The recent holistic proteomic analysis of Mladenov et al. [35] indicated the accumulation of multiple proteins associated with drought stress in *H. rhodopensis*: cell wall biosynthesis enzymes (e.g., UDP-apiose/xylose synthase, pectin methylesterase), mitochondrial transporters (e.g., mitochondrial outer membrane voltage-dependent anion channel), autophagy-associated elements (e.g., autophagy-associated gene proteins 3 and 9), oxidative stress protection (Cu/Zn-SOD), and protective proteins (20 kDa dehydrin, ELIP1), among others. Previously, Gechev et al. [36] reported the expression of HSP genes upon drought stress in *H. rhodopensis*. However, these studies have not specified the ecotype of the plants that were studied. Thus, natural variation of proteome-level protective mechanisms has not been revealed before.

Although Mladenov et al. [35] performed a detailed analysis of the proteome of *H. rhodopensis*, protein isolation and separation techniques may induce a selective loss of proteins [37]. Thus, here we applied a simple SDS-PAGE 1D separation of the leaf total proteome and analyzed approx. 48 and 14 kDa bands showing density changes upon desiccation. Among the de novo accumulating proteins, multiple polypeptides identified by Mladenov et al. [35] were also detected (e.g., ELIP, UDP-apiose/xylose synthase, pectin methylesterase). Generally, we found that an increased abundance of polypeptide bands on 1D SDS-PAGE primarily appeared in the molecular weight region below 55 kDa, thus proving to be smaller than RbcL. Since RbcL has the highest abundance in the leaf proteome (about 25% of total soluble proteins), its presence among the identified peptides is the result of proteolytic contamination. The identified protein bands showing altered density upon desiccation were in an identical molecular weight range both in sun and shade plants. Moreover, an increased band density was also found under low-temperature-induced dehydration—although, under low-temperature stress, leaves retained a typically higher RWC. These protein pattern changes also support the presence of identical mechanisms that enable drought- and low-temperature-induced desiccation tolerance in *H. rhodopensis* [11]. Cell rearrangement processes also proved to be identical under both drought- and low-temperature-induced desiccation [11,29]. Recovery of plants from desiccation induced by drought or low-temperature stress decreased the abundance of stress-accumulated proteins to the level of the well-hydrated control in both shade and sun plants, which further supports the existence of conserved protein accumulation mechanisms that are typical for desiccation in *H. rhodopensis*.

The drought- or low-temperature-induced desiccation brings about the rearrangement of the content of mesophyll cells in *H. rhodopensis*, leading to the formation of secondary vacuoles [11,29]. Although the solutes that are stored in these secondary vacuoles have not been fully understood yet, the massive accumulation of sucrose in leaves during drought-induced desiccation [29] suggests that they primarily function in balancing the osmotic conditions in the cells and thus contribute to the vitrification processes. In plants, vacuolar H^+^-ATPases (belonging to VHAs), localized in various members of the endomembrane system, are responsible for the acidification of the lumen bordered by these membranes, such as the vacuole. Moreover, they also maintain the homeostasis of multiple ions and metabolites by mediating active transport across the tonoplast, but also provacuoles [38]. VHAs also control sugar transport [39]. Multiple VHA subunits and subunit homologs have been reported to be enhanced by drought stress and desiccation so far [40,41,42]. Moreover, the overexpression of *Malus domestica* B-type VHA contributed to drought stress resistance [43]. VHA subunit H is a regulatory element that prevents the MgATPase activity of the dissociated V_1_ subunit [44,45]. Although subunit H has not been reported so far as a responsive element in drought stress tolerance, highly regulated solute transport and the proposed accumulation of sucrose and other molecules in the secondary vacuoles suggest a correlation with the increasing abundance of subunit H.

Vacuoles serve as the main cellular reservoirs for sugars [46,47]. Altered sugar metabolism upon desiccation has been indicated before [36,48,49] and was connected with the formation of secondary vacuoles [29]. However, the alterations in the carbohydrate metabolism also affect the biosynthesis and composition of cell wall carbohydrates [35]. Modification of the structure of cell wall polysaccharides plays a crucial role in the dehydration process in resurrection plants [34]. We identified multiple enzyme proteins that operate in the carbohydrate metabolism (galactose mutarotase-like/aldose 1-epimerase, UDP-D-apiose/UDP-xylose synthase, triosephosphate isomerase, pectin methylesterase, enolase) in desiccated leaves. The enhanced accumulation of the cytosolic triosephosphate isomerase that catalyzes the reversible interconversion of the triose phosphate isomers suggests the activation of core carbohydrate metabolism in the cytoplasm that could lead both to starch biosynthesis and to catabolic functions. Since during desiccation, starch granules disappear in the chloroplasts [29] and chloroplasts exchange triosephosphates and malate/oxaloacetate across the chloroplast envelope membrane, cytosolic triosephosphate isomerase seems to be linked to carbohydrate mobilization from the starch granules. Aldose 1-epimerase, which catalyzes the conversion of α and β anomers of hexoses, is a key enzyme in starch degradation [50]. Its enhanced accumulation also supports the primary importance of starch degradation during desiccation. However, aldose-1 epimerase-like enzymes were also shown to be interlinked with the operation of pectin methylesterase in stress control [51]. We also detected an increased presence of pectin methylesterase in desiccated samples. Enolase (phosphopyruvate hydratase) is also a cytoplasmic enzyme that converts 2-phosphoglycerate into phosphoenolpyruvate [52]. Enolase accumulation was reported in drought-tolerant wheat (*Triticum aestivum*) variety “Ningchun 47” [53]. *Arabidopsis* enolase locus *LOS2* was also reported to be important in cold stress tolerance [54]. The increased accumulation of enolase in *H. rhodopensis* also supports that catabolic functions are accelerated in the leaf cells upon desiccation.

Phosphoenolpyruvate, however, is also a precursor in the biosynthesis of phenolics in the chloroplasts [55]. Since the accumulation of phenolic compounds in the thylakoid lumen is significant during desiccation [25,56], at least a part of phosphoenolpyruvate biosynthesis is directed towards the chloroplast accumulation of phenolics. Besides cytoplasmic carbohydrate metabolism enzymes, the induction of the cell wall UDP-D-apiose/UDP-xylose synthase was also detected, which is similar to the results of Mladenov et al. [35]. UDP-D-apiose takes part in the biosynthetic pathway of the cell wall D-apiose. It cross-links rhamnogalacturonan II polysaccharides to form the pectin polysaccharide apiogalacturonan [57]. Moreover, apiose/xylose can also be linked to glucose residues of phenolic/phenylpropanoid glycosides in Gesneriaceae in a taxon-specific way [58,59]. In *Camellia* species, the enhanced UDP-D-apiose content was found to correlate with cold stress resistance [60]. The importance of the accumulation of phenolics is further supported by the detection/increased accumulation of the 55 kDa hypothetical protein/81E8-like cytochrome P450 monooxygenase in desiccated samples. 81E subfamily cytochrome p450 enzymes operate in the specific hydroxylation of phenylpropanoid isoflavones [61,62]. Since among *H. rhodopensis* metabolites, among others, 2′-hydroxyflavanone, 7-hydroxyflavanone, 4′-methoxyflavanone, 5-methoxyflavanone, and hispiduline (monomethoxyflavone) glycosides are abundant compounds [63,64], the biosynthesis and accumulation of hydroxylated (iso)flavone derivatives indicate their role is desiccation tolerance.

In the untargeted proteome analysis, we also identified a 15 kDa protein that accumulated during desiccation in *H. rhodopensis* leaves. Reverse blasting approved its homology to 1^VOC^ proteins. The function of any close homologs of the *H. rhodopensis* protein has not been identified yet, but the accumulation of DSI-1^VOC^ protein was previously reported in *Xerophyta humilis*, a South African poikilochlorophyllous resurrection plant, upon desiccation [65]. Although the function of the DSI-1^VOC^ protein has not been clarified, it might function in the detoxification of methylglyoxal, a by-product of triosephosphate breakdown, threonine catabolism, and acetone detoxification [65]. DSI-1^VOC^ was also reported to share homology with glyoxalase I but lack glyoxalase activity [65]. The induction of 1^VOC^ proteins in response to drought stress was also reported in *Brassica napus*, where it was suggested to function in the protection of embryos of high oil content under drought [66]. VOCs were also suggested to be related to the protection of the lipid metabolism [67]. Methylglyoxal is a toxic molecule that affects the redox status of the cells [68,69]. Thus, the maintenance of the redox status is of primary importance. Previously, we have indicated that the accumulation of malondialdehyde is different in shade and sun plants [70,71], suggesting ecotype-level differences in antioxidative protection. Similar to the results of Mladenov et al. [35], here we also identified the increased activity of SODs that play a role in the elimination of superoxide anion radicals [72]. Nevertheless, besides the increased abundance/activity of chloroplast Cu/ZnSOD III that was also reported by Mladenov et al. [35], we also identified the activation of FeSOD in sun plants that reflects ecotype-level variations in the antioxidative protection. FeSOD is an ancient type of SOD with a prokaryotic origin that is exclusively located in the chloroplasts in higher plants [73]. The activation of chloroplast SODs indicated that the elimination of superoxide anion radicals is predominant in the plastids. Since this induction is even higher in the sun ecotype, the formation of superoxide anion radicals is linked to the functioning of the photosynthetic apparatus and the increased prevalence of PSI and PSI+LHCII supercomplexes in sun plants [9]. Moreover, the higher enhancement in the SOD activity in sun plants during desiccation can also be associated with the previously reported higher Rubisco abundance, CO_2_ assimilation rate, and PSI activity in the sun plants compared to the shade ones [29,30]. Another part of the reactive oxygen species could originate from the excess light absorption of the chlorophyll molecules, especially if the photosynthetic chlorophyll-protein complexes do not function. ELIPs are LHC-like chlorophyll *a*/*b*-binding proteins functioning in photoprotection by binding chlorophylls of photosynthetic proteins targeted to degradation [74]. Similar to multiple studies before, we also detected the accumulation of ELIPs upon desiccation; thus, the accumulation of ELIPs seems to be a general response to drought-induced desiccation [30,75,76].

During desiccation, cellular proteins also become vulnerable. HSPs protect cell proteins from the detrimental effect of oxidative stress that plants suffered from during dehydration [77]. Gechev et al. [36] reported a constant high expression of HSPs indicating a constant primed status of *H. rhodopensis* for desiccation tolerance. Among HSPs, sHSPs contribute to the protection of membranes and proteins and act as molecular chaperones [78,79]. They are proteins of 12–42 kDa conserved across higher plants and act as ATP-independent molecular chaperones binding denatured proteins, thus preventing their irreversible aggregation. Accumulation of sHSPs was previously reported as a drought stress response [80,81]. In contrast to the results of Gechev et al. [36] we only detected sHSPs in sun but not in shade plants in the well-hydrated stage. In shade plants, sHSPs were only detected in severely dehydrated plants. Similar to the results of Gechev et al. [36], the constitutive expression of sHSPs was also reported in *D. hygrometricum* and *C. plantagineum* [82,83], whereas in multiple other taxa, the upregulation of sHSPs transcripts was found during dehydration [79,84]. Thus, we suppose that the priming effect primarily stands for the sun plants, whereas in the shade plants, even though the transcript of sHSPs would be constantly present, the protein accumulation is only triggered by drought stress conditions; thus, there is an ecotype/environmental condition-dependent variation in sHSPs.

Besides oxidative damage, proteins also become vulnerable upon desiccation due to the loss of water molecules. To protect the proteome, dehydrins, which are unstructured hydrophilic, thermostable proteins varying between 9.6 and 200 kDa in size, are accumulated [21]. Dehydrins are group 2 of late embryo-genesis abundant proteins [17,19] having chaperone-like functions in plant cells. Thus, they contribute to the protection of proteins but also function as ROS scavengers [21,22]. The accumulation of proteins such as dehydrins and sHSPs enhances plant tolerance to various stress factors [23]. Dehydrins are primarily important in resurrection plants during dehydration–rehydration cycles [22,85]. Comparing our results to literature data, we suggest that dehydrin band 5 corresponds to thylakoid localized dehydrin. Recently, it was demonstrated with two different anti-dehydrin antibodies that a dehydrin with an apparent molecular weight of ~20–22 kDa is a protein localized in the thylakoid membranes of *H. rhodopensis* [35,86]. In contrast to the results of Mladenov et al. [35], who reported a constant expression and thus net amount of both the phosphorylated and non-phosphorylated 20 kDa YSK_2_-type dehydrin, we found that the relative intensity of the approx. 20 kDa band shows ecotype-level differences. Although the pattern of protein accumulation in shade plants shares similarities with the results of Mladenov et al. [35], sun plants clearly accumulate a massive amount of dehydrins as a response to severe desiccation. Multiple studies demonstrated a positive correlation between the accumulation of dehydrins, soluble sugars, and enzymes related to sugar metabolism transcripts during desiccation [87,88]. Therefore, the accumulation pattern of dehydrins also supports the ecotype/environmental condition-dependent variation to desiccation.

Besides the protection of the proteins already synthesized, the enhanced protein biosynthesis also seems to be linked to the desiccation response in *H. rhodopensis*. In the untargeted proteome analysis, we revealed the increased abundance of two translation-related proteins, a chloroplast Tu-class elongation factor and a cytoplasmic 4A class initiation factor. IF-4A is involved in the binding of mRNA. It also performs duplex RNA helicase activity and relaxes duplexes in the 5′ untranslated region of eukaryotic mRNAs [89,90]. IF-4A is also involved in the operation of small non-coding RNA (sncRNA)-based translation control. The sncRNAs suppress the RNA helicase activity of IF-4A [91]. Overexpression of IF-4A results in an increased tolerance against various abiotic stresses [92], especially drought stress [93]. Since both oxidative stress and the loss of cellular water content affect the structure of RNAs, affecting RNA helicases also has a high potential in developing stress-resilient crops [94]. EFTu, a chloroplast protein translation component, also proved to be important in abiotic stress responses [95,96]. Moreover, ROS-mediated oxidation of EFTu is an important process that occurs under abiotic stresses [97]. Therefore, the increased abundance of both IF-4A and EFTu proteins contributes to the stabilization of the translation processes during leaf dehydration.

## 4. Materials and Methods

### 4.1. Plant Material and Experimental Design

Experiments were conducted on *Haberlea rhodopensis* Friv. plants originating from the Rhodope Mountains, South-West Bulgaria, 1000–1200 m a.s.l. region. Plants derived from sun-exposed limestone rocks (“sun” plants) received full sunlight (photosynthetic photon flux density (PPFD) of 1500–1700 µmol m^−2^ s^−1^ at midday in June) that results in a leaf-level air temperature of 30–37 °C and relative air humidity of approx. 15–30%. Plants derived from low irradiance conditions are understory plants growing in deeply shaded rock-crevice habitats (“shade” plants) exposed to a PPFD of approx. 25 µmol m^−2^ s^−1^ at midday in June that results in a leaf-level temperature of 21–25 °C and a relative humidity of 40–45%. Adult rosettes of similar size and appearance of well-hydrated (90% RWC) and desiccated plants with approx. 70, 50, 20, and 8% RWC were collected from their natural habitats, without damaging either the leaves or the roots, and were transferred to the laboratory. Experiments were conducted on fully expanded mature leaves of well-hydrated (90–95% RWC—90), moderately dehydrated (65–75% 45–55% RWC—70, 50), severely dehydrated (RWC 20), and dry plants (6–8% RWC—8) as well as after 1 day (50–60% RWC—R1) or 6 days (90–95% RWC—R6) of rehydration. Plants were rehydrated under laboratory conditions by watering them in a modified desiccator. The water at the bottom of the desiccator was pumped up, thus ensuring a permanent high humidity level. Samples were collected from dehydrated and rehydrated plants. Light intensity was measured using a QSPAR Quantum Sensor (Hansatech, Norfolk, UK), and leaf temperature and relative humidity values were measured using a Pocket Profi-Termohygrometer (TFA, Wertheim-Reicholzheim, Germany).

To acclimate plants to low-temperature conditions, plants were cultivated under natural light (PPFD of 1200–1500 µmol m^−2^ s^−1^ in summertime, and PPFD of around 100 µmol m^−2^ s^−1^ in wintertime) and shade (PPFD of around 30 µmol m^−2^ s^−1^) conditions in ex situ collection (Botanical Garden of Eötvös University, Budapest). Fully expanded mature leaves of sun and shade control (17–20 °C in September), cold-acclimated (5–6 °C, in November), freeze-stressed (10-day average temperature of approx. –3 °C, in January), and recovered (20–22 °C, in May) plants were collected. Light intensity was measured with the built-in photometer of an AP4 porometer (Delta-T Devices, Cambridge, UK). Environmental temperature was recorded using a minimum/maximum thermometer placed next to the experimental plant material.

### 4.2. Determination of Relative Water Content (RWC)

The RWC of leaves was determined gravimetrically. Fresh weights were recorded right at collecting, and saturated and dry weights were measured after saturating the water content by incubating leaf discs on wet filter paper overnight at 4 °C in the dark and after oven drying at 80 °C to a constant mass, respectively. RWC is expressed as the percentage of water content in dehydrated tissue compared to water-saturated tissues, using the following equation:

RWC (%) = (fresh weight − dry weight) × 100/(saturated weight − dry weight).

### 4.3. Extraction and SDS PAGE Separation of Leaf Proteins

Total leaf proteins were extracted in Laemmli [98] solubilizing buffer (62.5 mM Tris-HCl, pH 6.8, 2% (*w*/*V*) SDS, 2% (*w*/*V*) DTT, 8.7% (*w*/*V*) glycerol) and further solubilized at room temperature for 30 min. Samples containing about 10 µg proteins and 0.001% (*w/V*) bromophenol blue were applied per lane. Polypeptides were separated according to Laemmli [98] by applying 10–18% gradient polyacrylamide gels containing 8.7% (*w*/*V*) glycerol using a MiniProtean apparatus (BioRad, Hercules, CA, USA) with a constant current of 20 mA per gel at 6 °C for 2 h.

### 4.4. Protein Blotting and Western Blot Analysis

Leaf proteins separated by SDS-PAGE were transferred to Hybound-C Extra nitrocellulose membranes (Amersham Pharmacia Biotech., Piscataway, NJ, USA) using wet blotting systems (BioRad, Hercules, CA, USA). Transfer buffer containing 25 mM Tris, pH 8.3, 192 mM glycine, and 20% (*V*/*V*) methanol was used, and blotting was carried out at 4 °C using 90 V constant voltage (<0.4 A) for 3 h. Membranes were probed with primary antibodies against the lysine-rich domain segment of plant dehydrins (kind gift from Timothy J. Close, University of California, Riverside, USA) or α-crystallin domain of sHSPs (kind gift from Scott A. Heckathorn, University of Toledo, OH, USA). Horseradish peroxidase-conjugated goat anti-rabbit secondary antibody (170-6515, BioRad, Hercules, CA, USA) was used. The resulting bands were visualized by color reaction following the manufacturer’s instructions.

Densities of the lanes (sum protein in a sample) or the given polypeptide band (SDS-PAGE) or resulting bands (Western blot) were determined using Phoretix 4.01 software (Phoretix International, Newcastle upon Tyne, UK).

### 4.5. Activity Measurement of Superoxide Dismutase (SOD) Isoforms

The activity of SOD (EC 1.15.1.1) isoenzymes was measured according to Giannopolitis and Ries [99] with modifications. Leaf samples of 100 to 50 mg, depending on the water status, were homogenized on ice in 1 mL isolating buffer (50 mM Na-K-PO_4_ buffer, pH 7.0, 1.0 mM EDTA, 0.1% (*V*/*V*) Triton X-100, 5 mM Na-ascorbate, 2 mM PVP). The cell debris was pelleted by a 20,000× *g*, 20 min centrifugation. A clear fraction of the supernatant was collected as a crude extract. To separate SOD isoforms in the crude extract, a moderate solubilization was applied in 5 mM Tris-HCl, pH 6.8, 0.01% (*w*/*V*) SDS, 8.7% (*w*/*V*) glycerol, and 0.001% (*w*/*V*) bromophenol blue. Native proteins were separated on 10–18% gradient PAGE [98] supplying 0.01% (*w*/*V*) SDS to the cathode buffer only. Gels were stained for SOD activity in 50 mM Na-K-phosphate buffer, pH 7.8, 0.1 mM EDTA, 13 mM methionine, 60 μM riboflavin, and 2.25 mM Nitro Blue Tetrazolium. The gel was incubated in the staining solution for 15 min in darkness to achieve an equal penetration of the components. Riboflavin (Rbfl) was excited using a 250 W mercury lamp to generate superoxide anion radicals in the reaction of excited Rbfl to methionine. Activity-stained gels were scanned using an Epson Perfection V750 PRO gel scanner. Densitometry was performed in Phoretix 4.01 (Phoretix International, Newcastle upon Tyne, UK). SOD activity was normalized on the protein content of the samples. SOD isoenzymes were identified based on the selective inhibition (KCN sensitivity of Cu/ZnSODs and H_2_O_2_ sensitivity of both Cu/ZnSODs and FeSOD) results of Yahubyan et al. [32]. Total protein content of the crude extracts was determined by separating the proteins using SDS-PAGE and comparing the cumulative density of the Coomassie-stained bands with reference [100].

### 4.6. Protein Identification by Mass Spectrometry

The 1D SDS-PAGE polypeptide bands showing altered density upon desiccation of shade plants were subjected to untargeted proteomic determination. After reduction with dithiothreitol and alkylation with iodoacetamide, the proteins of the cut polypeptide bands were subjected to in-gel digestion by trypsin for 4 h at 37 °C. The tryptic digests were subjected to LC-MS/MS analysis using LCQ Fleet with an ion trap mass spectrometer coupled on-line with a nanoAcquity UPLC (Thermo Fisher Scientific, Waltham, MA, USA) using a 90 min long gradient.

Data analysis: The MS/MS peak list was subjected to database search. Since genomic data of the *H. rhodopensis* were not available in databases at the time of the analysis, a *D. hygrometricum* database (NCBI HabRho DORHY T10) supplemented with total Viridiplantaae BLAST was applied in the identification of the peptides (with the addition of pig trypsin) (Tables S1 and S2). Annotations were checked by running a BLAST search against flowering plant (taxID: 3398) refseq_RNA on NCBI (http://blast.ncbi.nlm.nih.gov/; accessed on 26 October 2022). Annotations were updated by running a BLASTX search in NCBI (https://blast.ncbi.nlm.nih.gov/Blast.cgi) accessed on 26 October 2022. Proteomic tool software ProteinProspector v6.4.2 (https://prospector.ucsf.edu/prospector/mshome.htm; accessed on 26 October 2022) was used. Parameters of the detailed search were as follows: Database: UniProt.Dorcoceras hygrometricum.random.concat/UniProt.Haberlea rhodopensis.random.concat (48439/48439 entries searched) and Viridiplantae UniProtKB.2020.09.02 (10293523/189525031 entries searched). Regarding the blast results, we primarily applied the *D. hygrometricum* annotations. In certain cases, where blasting against *D. hygrometricum* gave no hits, we applied the blast results against whole Viridiplantae; additional accession_numbers: 139429 (Porcin Tripsyn); const_mod: Carbamidomethyl (C); enzyme: TrypsinPro with maximum 2 missed cleavages; msms_parent_mass_tolerance: 0.2 Da; fragment_masses_tolerance: 0.8 Da; instrument_name: ESI-ION-TRAP-low-res; variable modifications: Acetyl (Protein N-term), Acetyl+Oxidation (Protein N-term M), Gln->pyro-Glu (N-term Q), Met-loss (Protein N-term M), Met-loss+Acetyl (Protein N-term M), Oxidation (M). The molecular weights of the predicted proteins were analyzed. Predicted proteins significantly (15%—taking into account the width of the cut band) smaller or larger than the apparent mid-band molecular weight of the polypeptide band (calibrated according to Sigma molecular weight standards: bovine serum albumin (66 kDa), ovalbumin (45 kDa), glyceraldehyde-3-phosphate dehydrogenase from rabbit muscle (36 kDa), carbonic anhydrase (29 kDa), trypsinogen from bovine pancreas (24 kDa), soybean trypsin inhibitor (20.1 kDa), α-lactalbumin, bovine milk (14.2 kDa), MW calibration performed in Phoretix 4.01) were generally excluded from the analysis (aggregation/degradation products). Proteins of larger MW than the mid-band molecular weight of the bands were kept as fragments. Comparative proteomics was performed using the polypeptides identified in the well-hydrated and desiccated samples. Fragments were also involved in comparative proteomics. To validate the hits, reverse protein blasting was performed using the best hit *D. hygrometricum* sequences in NCBI (http://blast.ncbi.nlm.nih.gov/Blast.cgi; accessed on 24 November 2022).

### 4.7. Statistical Analyses

Isolation of leaf proteins was repeated two times, sampling from pooled leaves of three different plants per treatment in two subsequent years. To compare means of stages, unpaired Student’s tests and, for multiple stages, multifactor ANOVA analyses with Tukey–Kramer multiple comparison post hoc tests and Fisher least significant difference tests were performed using InStat v. 3.00 (GraphPad Software, San Diego, CA, USA) and Statgraphics Plus v 5.1 (Statgraphics, The Plains, VA, USA), respectively. The term “significantly different” means that the probability of similarity of samples is *p* ≤ 0.05.

## 5. Conclusions

Maintenance of redox homeostasis and the integrity of the biochemical processes have primary importance in resurrection plants surviving cell desiccation. Moreover, homoiochlorophyllous resurrection plants also have to ensure that control over chloroplast-born ROS production is maintained. Although desiccation induced by low temperature and drought stress triggered similar responses in the protein pattern, the natural variation of these responses calls attention to the pre-conditioning/priming effects that have high importance in the desiccation responses and also in the successful recovery. While shade plants showed a slightly higher amount of total accumulated polypeptides during dehydration, sun plants had more pronounced SOD activity and a greater abundance of dehydrins and sHSPs. Although previous holistic studies have shown the most important transcriptome- and proteome-level alterations in *H. rhodopensis* upon desiccation, we revealed further representatives of the desiccation-induced members of the proteome.

## Figures and Tables

**Table 1:** Proteins identified in Band 1 (48 kDa) and Band 8 (14 kDa) by LC-MS/MS analysis from desiccated (8% RWC) shade *H. rhodopensis* plants that are not present in controls.

Band	ACC#	Species	Protein Name	MW/kDa	No. of Peptides	% Cov
#1	KZV21119.1	*Dorcoceras hygrometricum*	Enolase	47	3	11.3
	KZV25501.1	*Dorcoceras hygrometricum*	UDP-D-apiose/UDP-D-xylose synthase 2	43	3	11.8
	KZV20320.1	*Dorcoceras hygrometricum*	V-type proton ATPase subunit H	53	1	2.8
	KZV34753.1	*Dorcoceras hygrometricum*	Hypothetical protein F511_00655 (elongation factor Tu, chloroplastic)	52	2	6.7
	KZV24609.1	*Dorcoceras hygrometricum*	Hypothetical protein F511_06435 (cytochrome P450)	55	1	3.9
	KZV18901.1	*Dorcoceras hygrometricum*	Hypothetical protein F511_12006 (initiation factor 4A)	47	2	7.0
#8	KZV14262.1	*Dorcoceras hygrometricum*	Triosephosphate isomerase	23	2	13.9
	KZV28384.1	*Dorcoceras hygrometricum*	Early light-induced protein	20	1	7.3
	KZV47616.1	*Dorcoceras hygrometricum*	Hypothetical protein F511_12885 (desiccation-induced 1VOC superfamily)	15	1	11.1
	Q27U82	*Eucalyptus globulus*	Pectin methylesterase (fragment)	11	2	18.4
	A0A2Z7AJ43	*Dorcoceras hygrometricum*	Galactose mutarotase-like superfamily protein isoform 1 (fragment)	36	1	4.8

## Data Availability

All datasets are contained within the article and in the supplementary material.

## Supplementary Materials

The following supporting information can be downloaded at: https://www.mdpi.com/article/10.3390/plants12020401/s1, Figure S1: SDS-PAGE pattern of total leaf polypeptides of *H. rhodopensis* shade (A) and sun (B) plants under cold acclimation followed by recovery; Figure S2: Representative densitograms of leaf total polypeptide PAGE patterns in shade ecotypes of *H. rhodopensis* plants; Figure S3: Representative superoxide dismutase (SOD) in-gel activity staining of native soluble proteins extracted from *H. rhodopensis* shade and sun plants and separated on 10–18% gradient gels; Figure S4: Polyacrylamide gel electrophoretogram of total leaf proteins (A,B) and dehydrin western blot (C,D) patterns of shade (A,C) and sun (B,D) *H. rhodopensis* plants; Figure S5: Polyacrylamide gel electrophoretogram of total leaf proteins (A,B) and sHSP western blot (C,D) patterns of shade (A,C) and sun (B,D) *H. rhodopensis* plants; Figure S6: Polyacrylamide gel electrophoretogram of total leaf proteins *of H. rhodopensis* (A) and bands processed for LC MS/MS analysis (B).
