# Supplementary material for: Protein Changes in Shade and Sun Haberlea rhodopensis Leaves during Dehydration at Optimal and Low Temperatures
Source: Plants (Basel). 2023 Jan 15;12(2):401. doi: 10.3390/plants12020401 (PMC9861795; doi:10.3390/plants12020401)
Supplement: Supplementary file 1 [file plants-12-00401-s001.zip › Supplementary data.pdf]

Supplementary data

Protein changes in shade and sun *Haberlea rhodopensis* leaves during dehydration at optimal and low temperatures

Gergana Mihailova [1,*,†], Ádám Solti [2,*,†], Éva Sárvári [2], Éva Hunyadi-Gulyás [3] and Katya Georgieva [1]

1 Institute of Plant Physiology and Genetics, Bulgarian Academy of Sciences, Acad. Georgi Bonchev Str., Bl. 21, 1113 Sofia, Bulgaria
2 Department of Plant Physiology and Molecular Plant Biology, Institute of Biology, Eötvös Loránd University, Pázmány P. sétány 1/C, H-1117 Budapest, Hungary
3 Laboratory of Proteomics Research, Biological Research Centre, Eötvös Loránd Research Network, Temesvári krt. 62., Szeged H-6726, Hungary
* Correspondence: gmihailova@bio21.bas.bg, mihailova.gergana.k@gmail.com (G.M.); adam.solti@ttk.elte.hu (Á.S.)
** G.M. and Á.S. contributed equally to this work and should be considered as first authors.

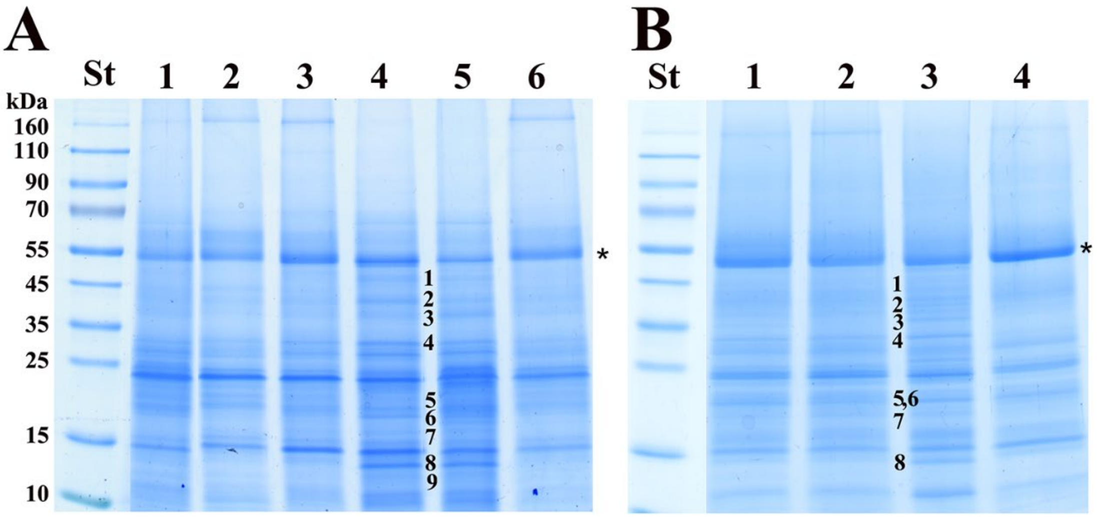

	1	2	3	4	5	6		1	2	3	4
°C (mean)	**17.6**	**6.4**	**2.0**	**-1.9**	**-2.3**	**13.6**		**17.6**	**6.4**	**-1.9**	**22.0**
precip. (mm)	7	21	0	9+snow	0	19		7	21	9+snow	15
RWC	**94.9**	**97.2**	**62.7**	**55.5**	**49.9**	**66.9**		**94.4**	**96.9**	**62.4**	**72.8**
SD	*3.4*	*5.1*	*7.3*	*10.4*	*9.9*	*1.2*		*3.6*	*3.8*	*4.7*	*6.0*

Figure S1. SDS-PAGE pattern of total leaf polypeptides of *H. rhodopensis* shade (A) and sun (B) plants under cold acclimation followed by recovery. Coomassie stained polypeptide patterns of leaves under control circumstances (lane 1), cold (lanes A2,3 and B2) and freezing temperature (lanes A4,5, B3), and following their recovery (lanes A6, B4). Approx. 10 µg protein was applied per lanes. St: Fermentas Page Ruler Prestained Protein SM0671 (Thermo Fisher Scientific) standards. Table inset indicates the environmental temperature (mean temperature of the week before sample collection) and the precipitation (cumulative total rainfall in mm during one month before sample collection) data. RWC values of the samples are given in order to address data to drought induced desiccation. Asterisks (*) on A and B show the position of Rubisco large subunit on the gels.

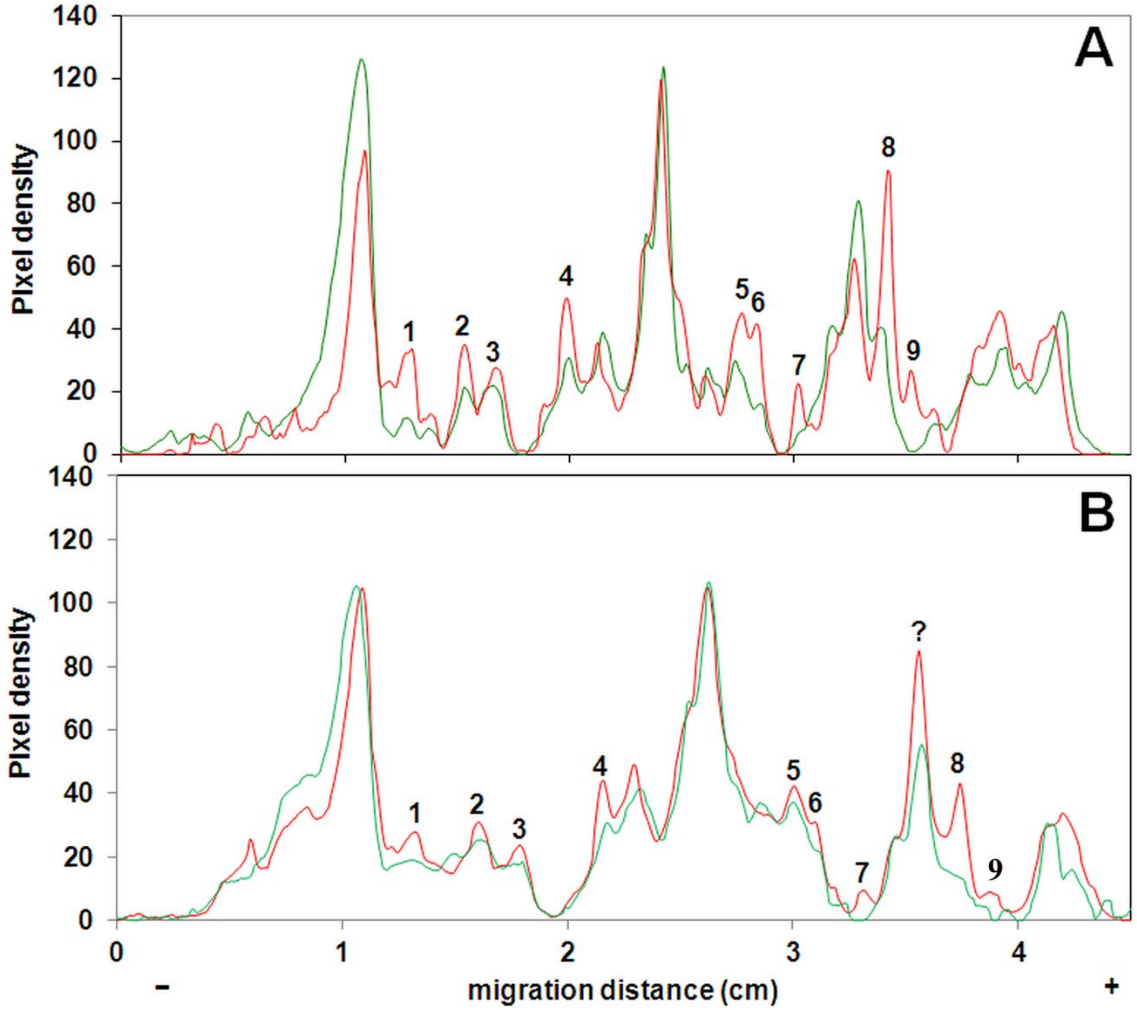

Figure S2. Representative densitograms of leaf total polypeptide PAGE patterns in shade ecotypes of *H. rhodopensis* plants. Elevation of bands (numbered 1 to 9) by drought induced desiccation to 8% RWC (A) and by temperature drop to a negative value (B). Control – green, dehydrated – red.

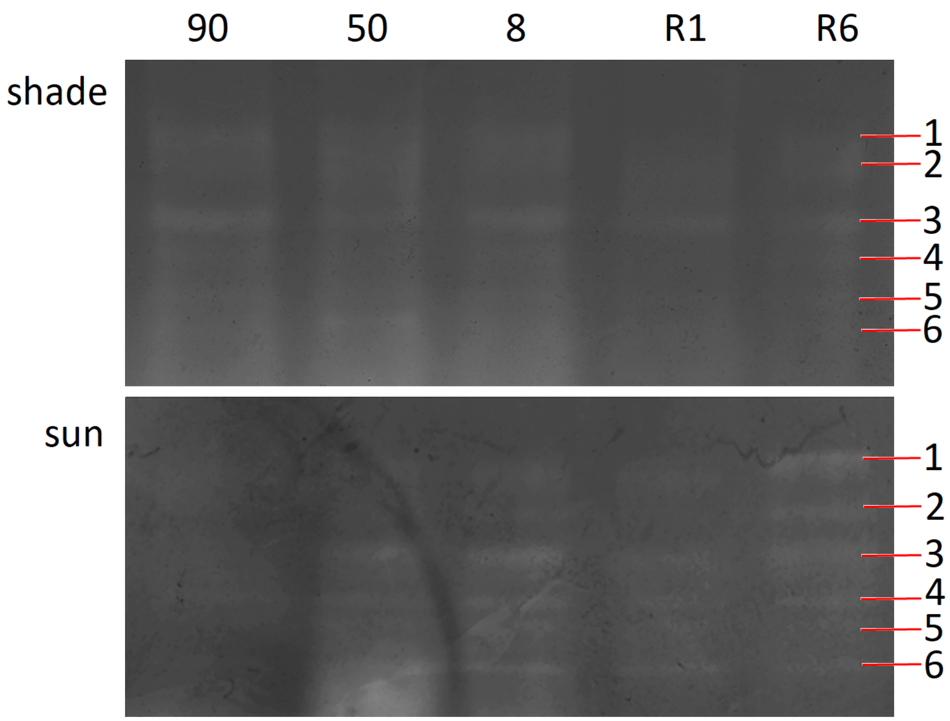

Figure S3. Representative superoxide dismutase (SOD) in-gel activity staining of native soluble proteins extracted from *H. rhodopensis* shade and sun plants and separated on 10–18% gradient gels. Samples were isolated from controls (90% RWC), different stages of desiccation (50 and 8% RWC) and after rehydration (1 and 6 days, R1 and R6, respectively). Six bands were detected that were identified as 1 – MnSOD; 2 – Cu/ZnSOD I.; 3 – FeSOD; 4 – Cu/ZnSOD II.; 5 – Cu/ZnSOD III.; 6 – Cu/ZnSOD IV.

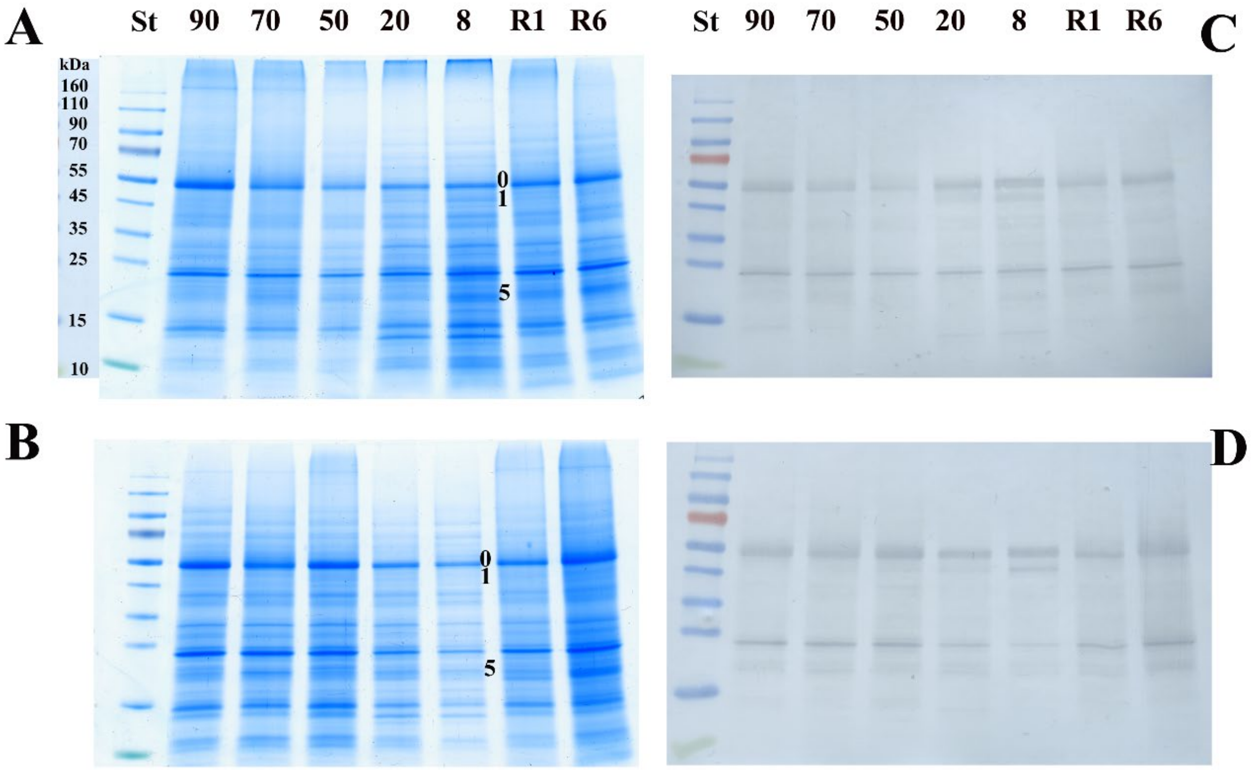

Figure S4. Polyacrylamide gel electrophoretogram of total leaf proteins (A, B) and dehydrin western blot (C, D) patterns of shade (A, C) and sun (B, D) *H. rhodopensis* plants. St: Fermentas Page Ruler Prestained Protein SM0671 (Thermo Fisher Scientific).

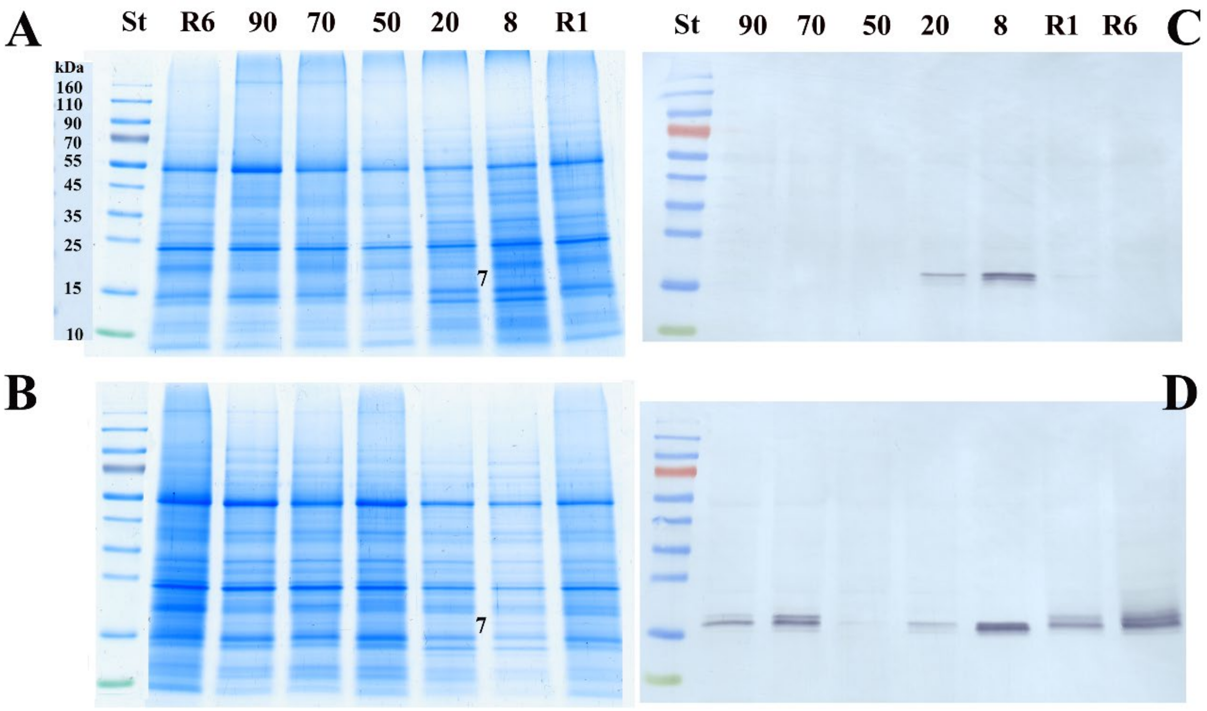

Figure S5. Polyacrylamide gel electrophoretogram of total leaf proteins (A, B) and sHSP western blot (C, D) patterns of shade (A, C) and sun (B, D) *H. rhodopensis* plants. St: Fermentas Page Ruler Prestained Protein SM0671 (Thermo Fisher Scientific).

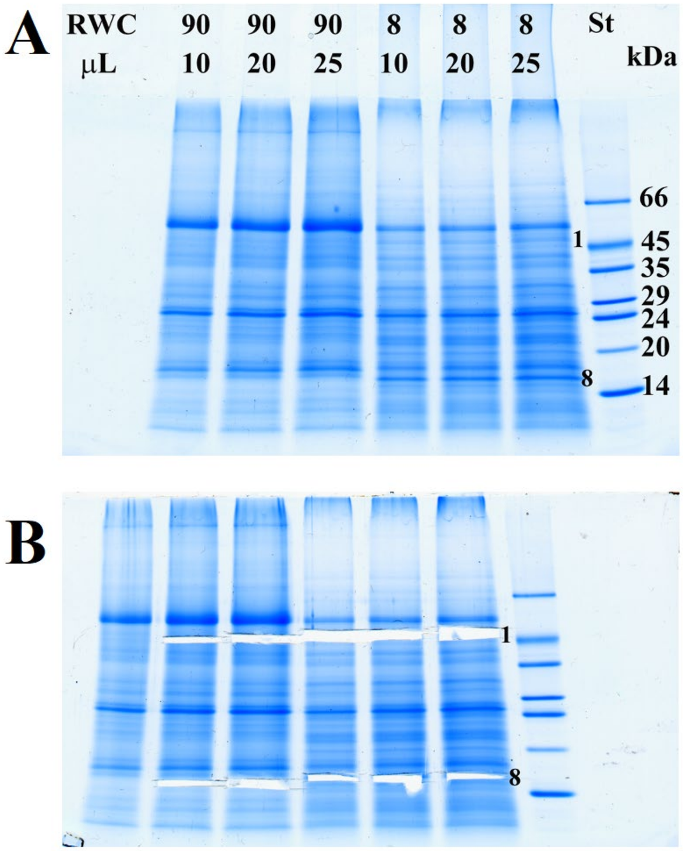

Figure S6. Polyacrylamide gel electrophoretogram of total leaf proteins *of H. rhodopensis* (A) and bands processed for LC MS/MS analysis (B). St: protein standards (Sigma) are (in kDa): bovine serum albumin (66), ovalbumin (45), glyceraldehyde-3-phosphate dehydrogenase from rabbit muscle (36), carbonic anhydrase (29), trypsinogen from bovine pancreas (24), soybean trypsin inhibitor (20.1), α-lactalbumin, bovine milk (14.2).
